# A Genetic Screen Using the PiggyBac Transposon in Haploid Cells Identifies *Parp1* as a Mediator of Olaparib Toxicity

**DOI:** 10.1371/journal.pone.0061520

**Published:** 2013-04-25

**Authors:** Stephen J. Pettitt, Farah L. Rehman, Ilirjana Bajrami, Rachel Brough, Fredrik Wallberg, Iwanka Kozarewa, Kerry Fenwick, Ioannis Assiotis, Lina Chen, James Campbell, Christopher J. Lord, Alan Ashworth

**Affiliations:** 1 The Breakthrough Breast Cancer Research Centre, The Institute of Cancer Research, London, United Kingdom; 2 CRUK Gene Function Laboratory, The Institute of Cancer Research, London, United Kingdom; 3 Tumour Profiling Unit, The Institute of Cancer Research, London, United Kingdom; German Cancer Research Center, Germany

## Abstract

Genetic perturbation screens have the potential to dissect a wide range of cellular phenotypes. Such screens have historically been difficult in diploid mammalian cells. The recent derivation of haploid embryonic stem cells provides an opportunity to cause loss of function mutants with a random mutagen in a mammalian cell with a normal genetic background. We describe an approach to genetic screens that exploits the highly active *piggyBac* transposon in haploid mammalian cells. As an example of haploid transposon (HTP) screening, we apply this approach to identifying determinants of cancer drug toxicity and resistance. In a screen for 6-thioguanine resistance we recovered components of the DNA mismatch repair pathway, a known requirement for toxicity. In a further screen for resistance to the clinical poly(ADP-ribose) polymerase (PARP) inhibitor olaparib we recovered multiple *Parp1* mutants. Our results show that olaparib toxicity to normal cells is mediated predominantly via *Parp1*, and suggest that the clinical side effects of olaparib may be on target. The transposon mutant libraries are stable and can be readily reused to screen other drugs. The screening protocol described has several advantages over other methods such as RNA interference: it is rapid and low cost, and mutations can be easily reverted to establish causality.

## Introduction

Forward genetic screens, in which random mutations are generated and the resulting mutants screened for a phenotype of interest, are of great use in the analysis of gene function. These screens have been most productively applied in model organisms with short generation times, such as yeast, *C. elegans* and *Drosophila*. In mammals, cell culture systems provide one approach to producing enough random mutants to adequately sample all mammalian genes. However screening in most mammalian cell lines has a major drawback compared to whole organisms: there is no breeding strategy available to produce homozygous mutants from randomly-generated heterozygotes. Therefore loss-of-function screens have always been challenging, as the diploid mammalian genome often ensures that any introduced mutation in a gene is compensated for by another copy of the gene on the homologous chromosome. Thus, RNA interference has remained the method of choice for cell culture screens, despite the development of effective mammalian transposons and their success in whole-organism screens [1], [2], [3].

The recent rediscovery and stable culture of haploid mammalian cells promises to change this situation. Haploid mammalian cells have only one copy of each chromosome, so random mutations can directly cause loss-of-function phenotypes. Haploid mammalian cell lines include a near-haploid human chronic myeloid leukaemia cell line [4] and its partially reprogrammed derivative [5], and the more recently derived haploid mouse embryonic stem (ES) cells [6], [7].

Here, we have applied piggyBac transposon mutagenesis in haploid ES cells to generate large, stable libraries of mutants for genetic screens, a process we will refer to as haploid transposon (HTP) screening. We have tested the potential of this system by identifying known genetic determinants of resistance to the chemotherapeutic 6-thioguanine. A major application for genetic screening is in investigating genetic mechanisms of toxicity and resistance as part of drug development. We therefore applied HTP screening to investigate mechanisms of response to the Poly (ADP-Ribose) Polymerase (PARP) 1/2 inhibitor olaparib [8], [9]. Although early clinical trials have demonstrated sustained antitumoural responses to olaparib, dose-limiting myelosuppression is also observed [10]. It is not known whether olaparib-induced toxicity to normal cells is mediated by PARP1 or PARP2 inhibition or is alternatively an “off-target” effect caused by inhibition of other enzymes. We found that *Parp1* itself is required for olaparib toxicity in wild type mouse ES cells, and depletion of *PARP1* in human cells also caused olaparib resistance. Our results not only exemplify the potential of HTP screens but also support a mechanism of action of PARP inhibitors in which the inhibited PARP1 enzyme forms a toxic DNA lesion.

## Results

**Figure 1:**
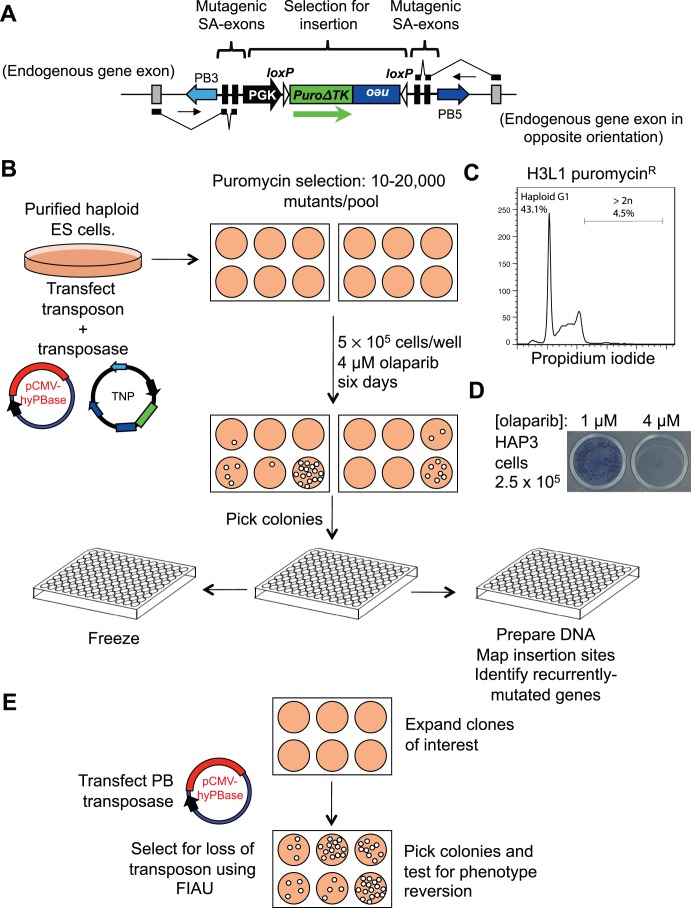
Generating and screening haploid transposon mutant libraries. **A.** The piggyBac transposon used for mutagenesis. The transposon cargo contains splice acceptors that disrupt transcription, but gene trapping is not directly selected for. PuroΔTK, is a positive-negative selection marker: puromycin can be used to select for integrations, and FIAU to select for loss of the transposon [30]. **B.** Outline of the mutagenesis and screening process. A detailed protocol is provided in Protocol S1. **C.** The mutant pool remains predominantly haploid, as shown by propidium iodide staining of fixed cells from library H3L1. **D.** Determining drug concentration for screening. Olaparib was used at a concentration that kills >2.5×10^5^ wild type haploid cells (4 µM). **E.** Scheme for further analysis of clones of interest by transposon reversion.

We designed a workflow and methods (Figure 1 and Protocol S1) that facilitate the mutagenesis and screening of HAP-3 ES cells [6] using a piggyBac transposon construct (TNP) designed to disrupt transcription and give wide genome coverage [11] (Figure 1A, B). This vector contains a positive-negative selection marker gene, *puro*Δ*TK*, driven by the mouse phosphoglycerate kinase promoter (PGK), which allows selection for insertion into the genome using puromycin. Mutagenesis is achieved by two pairs of splice-acceptor bearing exons at either end of the transposon cargo in opposite orientations, which should disrupt splicing if the transposon is inserted into an intron of a gene. The penultimate exon of each pair is engineered with several premature stop codons in each reading frame, which should cause degradation of fusion transcripts by the nonsense-mediated mRNA decay pathway, thus even insertions at the 3′ end of genes are likely to be mutagenic.

Using conditions that give, on average, one transposon insertion per cell [12], we mutagenised haploid HAP-3 ES cells to generate a mutant library, H3L1, containing 5–10,000 mutants (Figure 1B). To assess ploidy of the mutant population, we stained fixed cells with propidium iodide and analysed DNA content by flow cytometry. The library remained mainly haploid after puromycin selection (fewer than 5% cells with greater than 2C DNA content), indicating that most insertions occurred in haploid cells and should therefore cause loss of function mutations when inserted into genes (Figure 1C).

To test the extent of loss-of-function mutagenesis in the libraries, we used 6-thioguanine selection. This positive selection screen has been previously used as a benchmark for mutagenesis systems, as 6-thioguanine is known to require the DNA mismatch repair pathway for toxicity [13], [14]. We exposed the H3L1 library to 6-thioguanine, selected resistant colonies and mapped the genomic position of transposon insertion sites. Among the 18 clones sampled from the resistant population in which the insertion site could be mapped, we identified mutations in the DNA mismatch repair pathway genes *Msh2, Msh6* and *Mlh1* (Table 1). Importantly, three of the four expected mismatch repair genes were recovered (the exception being *Pms2*) from this library, including three different insertions in *Msh2*, indicating good genome coverage even in this sub-saturating library. This demonstrated that HTP screens can efficiently identify known mechanisms of drug toxicity.

**Table 1:** Insertion sites mapped in 6-thioguanine resistant mutants from library H3L1.

Chromosome	Position	Gene	No. of clones
17	87,679,642	*Msh2*	7
17	87,693,511	*Msh2*	1
17	87,677,541	*Msh2*	1
17	87,989,839	*Msh6*	1
9	111,277,467	*Mlh1*	1
14	59,984,872	*D14ERtd668e*	1
4	136,001,187	*Htr1d*	1
9	19,825,492	*Olfr866*	1
9	112,130,053	*Arpp21*	1
18	12,949,303	*Osbpl1a* (upstream)	1
16	70,988,785	No gene	1
14	105,125,079	*Rbm26*	1

To further exemplify the potential of HTP screens, we used the same conditions to generate 12 additional mutant libraries to obtain better genome coverage, each containing 10–20,000 mutants based on the number of puromycin-resistant colonies obtained (H3L2–H3L13, Figure 1B). We then exposed these to the PARP1/2 inhibitor olaparib at a concentration that results in a surviving fraction (SF) of <1 in 2.5×10^5^ wild type cells (Figure 1D).

**Figure 2:**
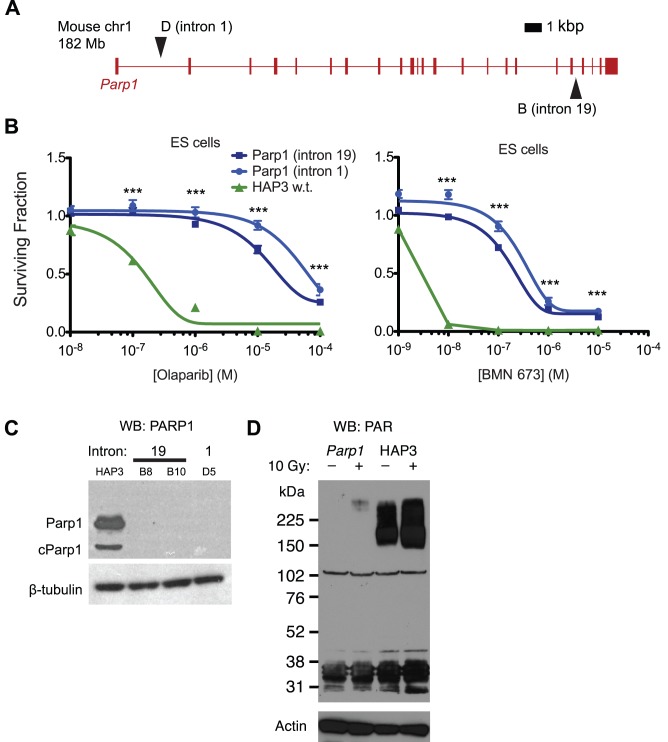
*Parp1* null mutants are resistant to PARP inhibitors. **A.** Positions of insertions in the *Parp1* gene in resistant mutants. **B.** Olaparib and BMN 673 dose-response curves for *Parp1* mutants (blue) and wild type HAP-3 cells (green). Surviving fraction is assessed using CellTiterGlo and normalised to DMSO-treated control cells. The mean of five replicates is shown; error bars show SEM. ***, *P*<0.001 for intron one mutant (D) compared to HAP-3. **C.** The *Parp1* mutations are null alleles. Lysates from the indicated cells were probed with anti-PARP or anti-β-tubulin antibodies. A lower molecular weight band corresponding to cleaved Parp1 is also detected in wild type cells at the long film exposure used here. Similar results were obtained using a different antibody and independently prepared lysates (see Figure 5A). **D.** Low PAR levels and decreased induction of PAR in response to ionising radiation in *Parp1* mutant ES cells. Data shown are representative of three experiments, using multiple *Parp1* mutant subclones and wild type cell lines.

Thirty-two colonies survived the olaparib treatment, and the insertion site was mapped in 24 using Splinkerette PCR. Unexpectedly, 18 colonies had insertions in *Parp1*, with two different *Parp1* insertion sites being independently detected in different mutant pools (Figure 2A and Table 2). Finding the same gene disrupted by different transposon insertions is strong evidence that the gene is required for sensitivity and the phenotype is not due to a background mutation unlinked to the transposon. As piggyBac preferentially reintegrates close to the site of excision when it transposes [15], finding the *Parp1* transposition events in multiple libraries confirms that these insertions arose independently and not from a secondary transposition event that reintegrated elsewhere in the same gene.

**Table 2:** Insertion sites mapped in olaparib-resistant mutants from libraries H3L2–H3L13.

Clone no.	SF_50_ (µM)	Library	No. of Clones	Chr	Position	Gene	Other sites
B4, B8, B10 (e.g.)	50	7	13	1	180,598,540	*Parp1* (intron 19)	8∶117,823,558
D5 (e.g.)	50	13	5	1	180,571,878	*Parp1* (intron 1)	
B1	5	2	1	2	73,943,760	u/s of *Atp5g3*	1∶195,241,859
B6	0.5	5	1	10	95,367,472	u/s of *Cradd*	
B9	10	7	1	15	50,903,588	u/s/of *Trps1*	
C2	>10	7	1	13	107,732,892	*Zswim6*	
C12	5	9	1	16	67,488,482	*Cadm2*	
B3	>20	5	1	2	6,643,847	*Celf2*	

**Figure 3:**
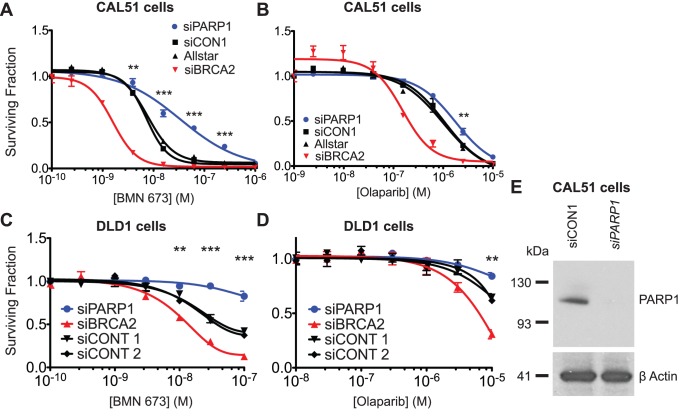
*PARP1* depletion by siRNA causes PARP inhibitor resistance in human cells. CAL51 (**A, B)** or DLD1 **(C, D)** cells were transfected with the indicated siRNAs and treated with BMN 673 **(A, C)** or olaparib **(B, D)**. Cell survival assayed using CellTiter Glo and expressed as a proportion of DMSO-treated control is shown on the y-axes. Allstar, siCONT1, siCONT2 are non-targeting siRNA controls. siRNA targeting *BRCA2* is included as a positive control known to sensitise cells to PARP inhibition [8]. The mean of five replicates is shown; error bars show SEM. **, *P*<0.01; ***, *P*<0.001 for *PARP1* compared to non-targeting control (two-way ANOVA with Bonferroni post test). **E** Confirmation of knockdown of PARP1 protein. Lysates from CAL51 cells transfected with the indicated siRNAs were probed with anti-PARP1 (top) or anti-β-actin (bottom).

*Parp1* mutants were 100-fold more resistant to olaparib than wild type cells (Figure 2B) and also showed profound resistance to another clinical PARP1 inhibitor, BMN 673 (Figure 2B). Prior to this analysis, we expected that genetic inhibition of *Parp1*, a major target of olaparib, would add to the growth inhibitory effect of this drug and thus resistance could be caused by an increase in Parp1 expression or activity. However, both *Parp1* mutants lacked detectable Parp1 protein when assayed by western blot (Figure 2C), suggesting that the transposon mutations most likely generate null alleles that ablate protein expression. *Parp1* mutant cells had greatly reduced levels of baseline and radiation-induced poly(ADP-ribose) (PAR), the product of PARP enzymatic activity, further suggesting that there is no active truncated protein expressed (Figure 2D, note that most PAR polymerisation after DNA damage occurs on Parp1 itself). The effect of *Parp1* ablation was neither mouse nor ES cell-specific, as silencing of *PARP1* by short interfering RNA (siRNA) in human CAL51 and DLD1 tumour cells also caused resistance to BMN 673 and olaparib (Figure 3).

**Figure 4:**
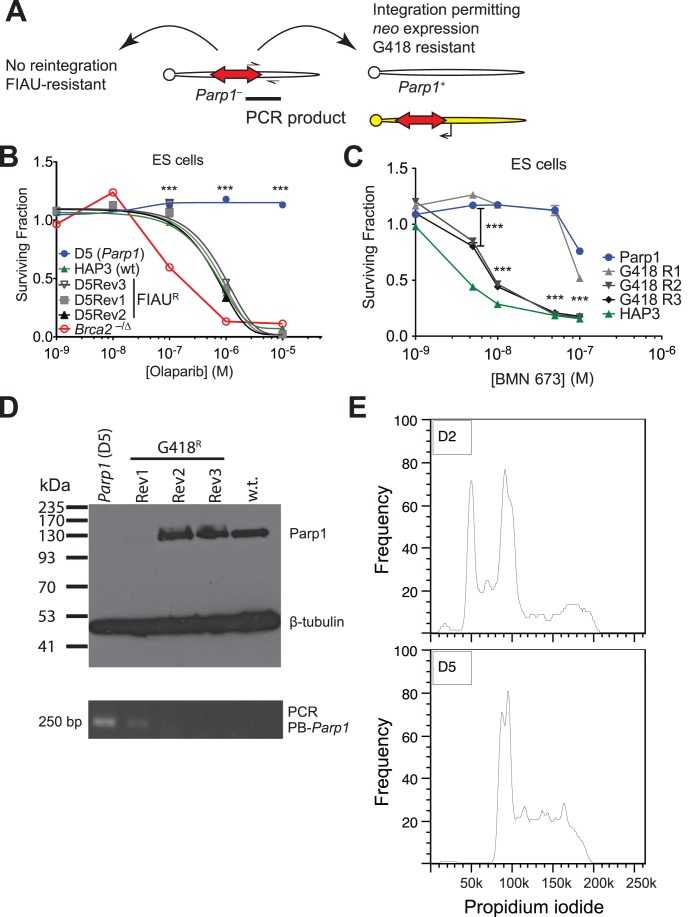
Reversion analysis of *Parp1* mutants. **A.** Schemes used for isolation of revertants from the D5 (intron one) *Parp1* mutant ES cells. **B.** Three FIAU-resistant clones isolated after transfection of clone D5 with PB transposase (Rev1–3, grey and black) have regained sensitivity to olaparib similar to wild type cells (green). *Brca2*-deficient ES cells [8], which are sensitive to PARP inhibition, are shown for comparison. Error bars show SEM, *n = *5. ***, *P*<0.001; comparison shown for D5Rev1 and *Parp1* mutant. **C.** Dose response curve for BMN 673 for three clones isolated using the G418 selection scheme. *n = 5*; ***, *P*<0.001; comparison shown for G418 R2 and *Parp1* mutant. **D.** The G418 resistant clone that remains PARP inhibitor resistant (clone R1) is still a *Parp1* mutant. Lysates from the indicated cells were probed with anti-PARP followed by anti-β-tubulin. **E.** Clone G418 R1 still contains the original *Parp1* insertion. DNA from the indicated cells (same order as above) was analysed for the presence of the transposon-genome junction by PCR using the primers shown in **A**. **F.** Most clones isolated from the screen have a diploid DNA content. Three out of nine tested had a mixture of haploid and diploid cells similar to D2 (top, this clone also has the intron 1 *Parp1* insertion); all others were fully diploid including clone D5 (bottom).

A unique advantage of the piggyBac system, not found in screening systems utilising RNA interference, is that each transposon can be precisely excised by simply re-expressing transposase, allowing formal proof that the insertion causes the mutant phenotype. The transposon vector used here allows negative selection via the thymidine kinase gene, which causes cells to be sensitive to FIAU, facilitating isolation of cells that have lost the transposon after transposase expression (Figure 4A). To exemplify this property of the piggyBac system, we isolated FIAU-resistant clones from the intron 1 *Parp1* mutant after transposase transfection and showed that these had reverted to olaparib sensitivity (Figure 4B).

No FIAU-resistant colonies were obtained without transposase transfection, however to exclude potential contamination of the culture with wild type cells we also isolated revertants by another method. The transposon used contains a promoterless *neo* selectable marker in the opposite orientation to the *puro*Δ*TK* gene that was used for selection. Although the *neo* gene has no promoter, some integration sites may permit expression of *neo* and lead to G418 resistance (Figure 4A). Therefore we also transfected the *Parp1* mutant with transposase and selected in G418 for clones where the transposon had excised and reintegrated into such sites. Of three G418 resistant clones analysed, two had reverted to wild type sensitivity and restored Parp1 expression (Figure 4C, D). One further clone that survived G418 selection had not lost the transposon from the *Parp1* locus and remained PARP inhibitor resistant and Parp1 null (Figure 4C, D); this may have arisen from incomplete selection with G418.

The clone with the intron 19 insertion contained an additional transposon insertion (Table 2) and is thus more difficult to revert using FIAU selection for loss of the transposon, particularly since the cells have also become diploid and therefore will contain four copies of the transposon. Haploid cells grown at low density appear to have an increased chance of diploidisation, as most mutant clones isolated from the screen were fully diploid (Figure 4E). The increased DNA content could potentially affect the response to agents that affect DNA repair such as PARP inhibitors, however the reversion experiment above formally proves that the mutant phenotype is not due to diploidy.

**Figure 5:**
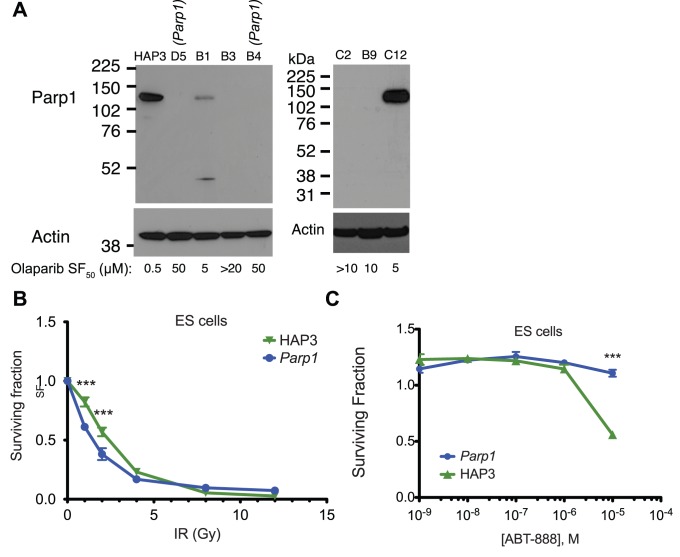
Other mutants isolated in the screen also lack Parp1 expression. **A.** Other clones isolated in the olaparib resistance screen, which did not revert, have lost Parp1 protein expression. Lysates from the indicated cells were probed with anti-PARP or anti-β-actin antibodies as indicated. The same result was obtained in an independent experiment with a different antibody. Olaparib SF_50_ was calculated from dose response curves as in (B); >10 means growth was >50% relative to untreated cells at the highest concentration tested (e.g. 10 µM). **B.** *Parp1* mutant ES cells are not resistant to ionising radiation. Cells were irradiated with the indicated dose one day after plating and assayed for growth five days later. **C.** *Parp1* mutant cells are also resistant to the PARP inhibitor ABT-888 (veliparib) at high doses. *n = *5, mean and SEM shown, ***; *P*<0.001.

Several mutants with insertions in genes other than *Parp1* were isolated in the screen, but no other genes had multiple different insertion events. Some mutants displayed similar olaparib sensitivity to wild type cells, indicating that they arose from incomplete selection, whereas others showed clonal olaparib resistance (Figure 5A). We were unable to isolate revertant clones from any of these mutants, suggesting that their olaparib resistant phenotype is unlikely to be linked to the transposon insertion. The most resistant mutants also lacked Parp1 protein expression, suggesting that Parp1 loss could also be the mechanism of resistance in these clones (Figure 5A). This could occur through spontaneous mutation or epigenetic inactivation at the *Parp1* locus or a regulator of *Parp1*– since the cells are haploid, the effects of any spontaneous mutations will be immediately apparent. Since all of the non-Parp1 mutants were only represented by a single colony, it is likely that these arise late in the culture, after the transposon mutagenesis step.

We also used transposon-directed insertion site sequencing (TraDIS) [16], a method to specifically isolate and sequence transposon-genome junctions using the Illumina Genome Analyser, to assess the mutants present in the H3L1–8 libraries prior to drug exposure (Table S1). Transposon-genome junctions from 48,707 positions were identified. In agreement with previous observations, 24,499 (50%) of these insertions were in genes. At least 8,841 different genes were mutated in these eight libraries, giving an average coverage of five insertions per mutated gene. However, the libraries will likely contain more mutants than this, as this sequencing experiment did not completely sample all the transposon insertions in the libraries. Most sites were only covered by a single read once PCR duplicates were removed and although both sides of the transposon were sequenced, few insertion sites were represented by sequences from both ends.

This sequencing identified insertions in the PARP superfamily genes *Parp6, Parp7, Parp9*, *Parp11*, *Parp12, Parp14* and *Parp16*; and the tankyrases *Tnks* (*Parp5a*) and *Tnks2* (*Parp5b*) (Table 3). As these genes were accessible to the transposon but were not selected by olaparib exposure it seems possible that these genes are not key determinants of resistance under these conditions.

**Table 3:** Transposon insertions in other PARP superfamily members identified in sequencing of insertion sites from H3L1–H3L8.

Gene	No. of insertion sites
*Parp6*	1
*Parp7* (*Tiparp*)	2
*Parp9*	4
*Parp11*	3
*Parp12*	1
*Parp14*	2
*Parp16*	2
*Tnks* (*Parp5a*)	6
*Tnks2* (*Parp5b*)	2

## Discussion

The unexpected observation that *PARP1* is the major genetic determinant of PARP1/2 inhibitor toxicity in normal cells suggests that PARP1 protein is a necessary component of the toxic DNA lesion. As autoparsylation of PARP1 is required for the release of PARP1 from sites of DNA damage [17], PARP inhibitors could prevent dissociation of PARP1 from DNA, causing a potentially lethal DNA lesion. In the absence of PARP1 expression, such lesions would not form and PARP inhibitor resistance could occur. This hypothesis is supported further by a recent study showing poisoning of PARP1 in the presence of olaparib [18].

Human cancer cell lines that have acquired resistance to the PARP inhbitor ABT-888 have also been previously shown to have reduced PARP1 levels, although these cells also acquired resistance to ionising radiation [19], which our *Parp1* mutant cells did not (Figure 5B). ABT-888 (also known as veliparib) was shown to trap PARP1 less efficiently than olaparib [18] and thus is not very toxic to wild type cells. In agreement with this result, our *Parp1* mutants were more resistant to ABT-888 than wild type cells, but high doses must be used to observe this (Figure 5C). Our results suggest that toxicity to normal cells in patients treated with PARP inhibitors may be an on-target effect mediated via PARP1 and therefore may not be reduced by developing more potent PARP inhibitors (Figures 2B and 3A, C). Furthermore, our results suggest the possibility that tumour-specific mutation or inhibition of *PARP1* could result in clinical PARP inhibitor resistance and disease progression.

The HTP screening system described here has several distinct qualities when compared to current mammalian genetic screening systems: it is not reliant on the purchase or synthesis of a reagent library (such as RNAi systems) and large mutant libraries can be rapidly generated using freely available plasmids. The 6-thioguanine screen was conducted in one well of a 6-well cell culture plate, and thus required far fewer cells compared with previous ES cell screening methods and gave a similar result in terms of recovering relevant mismatch repair mutants [20]. The transposon introduces a robust, heritable mutation, which may result in a better chance to observe a phenotype than with a siRNA knockdown that is unlikely to be complete nor to persist over a long period. This may be the reason why the olaparib resistant phenotype is not as pronounced in the human cell lines as in the mutant ES cells (Figure 3B, D). Indeed, despite numerous siRNA screens for modulators of olaparib response having been carried out, *PARP1* has not previously emerged as a resistance-causing hit by objective criteria.

After transposition, mutant libraries are stable and can be used for multiple screens, allowing common genetic mechanisms of resistance to be identified for different drugs. The reversion of transposition events allows causality to be established, a complex issue in RNAi screens where off-target effects are a major concern [21]. Even in the haploid system, reversion analysis is important for the investigation of mutants that show a phenotype, but for which no other independent insertions were recovered in the same gene. In the screen described here, none of the mutants for which we only recovered one colony reverted, and in fact most proved to have also lost Parp1 expression. Clones such as C12 (*Cadm2*) that have a mild resistance phenotype and retained Parp1 expression may have acquired another mutation that promotes resistance; however since the phenotype is not linked to the transposon insertion this is more difficult to investigate. Screening using a weaker selective pressure may result in more mutants with weaker resistance phenotypes and thus allow minor mechanisms to be investigated.

As demonstrated here and elsewhere [22], cells in culture accumulate mutations that can interfere with screening results where strong selection is applied. This is likely to be a particular problem when using haploid cells, since the effects of loss of function mutations will be immediately apparent. Thus cells should be cultured for the minimum time possible, and tested for the presence of resistant mutants prior to mutagenesis.

The high plating efficiency of ES cells at low density makes them ideal for positive selection screens as described here, but high-throughput sequencing methods for mapping transposon integrations [16] will further extend the scope of these screens, which are likely to be equally informative for investigating determinants of drug sensitivity.

## Materials and Methods

HAP-3 cells [6] were obtained from the laboratory of Anton Wutz (Cambridge Stem Cell Institute) and cultured on an SNL76/7 fibroblast feeder layer in 2i medium supplemented with LIF as previously described [23], [24]. After one passage on gelatin-coated plates to eliminate feeder cells, haploid cells were purified by fluorescence-activated cell sorting, staining with 20 µg/ml Hoechst 33342 for 20 minutes and using a BD FACS aria with an excitation wavelength of 407 nm (violet laser). For propidium iodide (PI) staining, cells were fixed overnight at −20°C in 70% ethanol, washed with PBS and incubated with 5 µg/ml PI and 80 µg/ml RNAseA for 15 minutes. For mutagenesis, ten million cells were transfected by electroporation (230 or 270 V, 500 µF; BioRad GenePulser) with 1 µg TNP transposon donor plasmid [11] and 10 µg pCMV-hyPBase transposase expression plasmid [25] and selected in 3 µg/ml puromycin for six days. To make the genome-wide library, each of three electroporations was plated on four separate plates to produce 12 sectored pools of mutant cells (H3L2–H3L13).

To isolate resistant mutants, 5–10×10^5^ cells were plated on a six-well feeder plate (this high density precludes isolation of *Hprt* mutants in 6-thioguanine due to cross-killing) and drugs added the following day. Drug concentrations used were: 6-thioguanine, 2 µM; olaparib, 4 µM; puromycin, 3 µg/ml. After isolation of mutants, ES cells were cultured in conventional ES cell medium supplemented with 15% fetal calf serum (FCS) and LIF. For survival curves, 2,500 ES cells were seeded per well on gelatinised 96-well plates. Drug-containing medium was added the next day (day two) and replenished 48 hours later. The live cell proportion was estimated on day six using CellTiterGlo (Promega).

To isolate revertants, mutant cells were electroporated with the hyPBase expression plasmid and cultured for three days without selection. Cells were then plated in 200 nM FIAU (1-(2-deoxy-2-fluoro-1-d-arabinofuranosyl)-5-iodouracil) or G418 (180 µg/ml). Untransfected cells were also plated in FIAU to ensure that the reversion was PBase-dependent and did not arise from spontaneous mutation or loss of the puΔTK selectable marker or from contaminating wild type cells in the culture.

Insertion sites in drug-resistant subclones were amplified using Splinkerette PCR as previously described [26] and mapped using iMapper [27]. We used the TraDIS method for large scale amplification and sequencing of insertion sites [16], which were mapped to mouse genome version NCBI m37 using bwa [28].

Antibodies used were: anti-PARP1 polycolonal (Cell Signaling #9542, Figure 2C, 3E), anti-PARP monoclonal C-2-10 (abcam ab105, Figure 4D, 5A), anti-PAR monoclonal 10H (Enzo 804-220-R100, Figure 2D).

Human CAL51 cells were obtained from DSMZ and cultured in DMEM supplemented with 10% FCS (Gibco), 2 mM L-glutamine and penicillin–streptomycin (Gibco). DLD1 cells were obtained from Horizon Discovery Ltd. and cultured in McCoy’s 5A medium with 10% FCS, 2 mM L-glutamine and penicillin–streptomycin. *Brca2* ^Δ/−^ ES cells have been previously described [29]. siRNA transfection was achieved using Lipofectamine 2000 (Invitrogen) in six-well format following the manufacturer’s instructions. All siRNAs were purchased from Dharmacon. Survival was assessed using CellTiter Glo after five days drug exposure.

## Supporting Information

Table S1 **A list of insertion sites identified by sequencing libraries H3L1–H3L8.**
(XLSX)

Protocol S1 **A detailed protocol for carrying out drug resistance screens.**
(DOCX)

## References

[1] Rad R, Rad L, Wang W, Cadinanos J, Vassiliou G, et al (2010) PiggyBac transposon mutagenesis: a tool for cancer gene discovery in mice. Science 330: 1104–1107. 20947725 10.1126/science.1193004 PMC3719098

[2] Vassiliou GS, Cooper JL, Rad R, Li J, Rice S, et al (2011) Mutant nucleophosmin and cooperating pathways drive leukemia initiation and progression in mice. Nature Genetics 43: 470–475. 21441929 10.1038/ng.796 PMC3084174

[3] Dupuy AJ, Akagi K, Largaespada DA, Copeland NG, Jenkins NA (2005) Mammalian mutagenesis using a highly mobile somatic Sleeping Beauty transposon system. Nature Cell Biology 436: 221–226. 10.1038/nature03691 16015321

[4] Carette JE, Guimaraes CP, Varadarajan M, Park AS, Wuethrich I, et al (2009) Haploid Genetic Screens in Human Cells Identify Host Factors Used by Pathogens. Science 326: 1231–1235. 19965467 10.1126/science.1178955

[5] Carette JE, Raaben M, Wong AC, Herbert AS, Obernosterer G, et al (2011) Ebola virus entry requires the cholesterol transporter Niemann-Pick C1. Nature 477: 340–343. 21866103 10.1038/nature10348 PMC3175325

[6] Leeb M, Wutz A (2011) Derivation of haploid embryonic stem cells from mouse embryos. Nature 479: 131–134. 21900896 10.1038/nature10448 PMC3209452

[7] Elling U, Taubenschmid J, Wirnsberger G, O’Malley R, Demers S-P, et al (2011) Forward and Reverse Genetics through Derivation of Haploid Mouse Embryonic Stem Cells. Cell Stem Cell 9: 563–574. 22136931 10.1016/j.stem.2011.10.012 PMC4008724

[8] Farmer H, McCabe N, Lord C, Tutt A, Johnson D, et al (2005) Targeting the DNA repair defect in BRCA mutant cells as a therapeutic strategy. Nature 434: 917–921. 15829967 10.1038/nature03445

[9] Bryant HE, Schultz N, Thomas HD, Parker KM, Flower D, et al (2005) Specific killing of BRCA2-deficient tumours with inhibitors of poly(ADP-ribose) polymerase. Nature 434: 913–917. 15829966 10.1038/nature03443

[10] Fong PC, Boss DS, Yap TA, Tutt A, Wu P, et al (2009) Inhibition of poly(ADP-ribose) polymerase in tumors from BRCA mutation carriers. New England Journal of Medicine 361: 123–134. 19553641 10.1056/NEJMoa0900212

[11] Huang Y, Pettitt SJ, Guo G, Liu G, Li MA, et al (2012) Isolation of homozygous mutant mouse embryonic stem cells using a dual selection system. Nucleic Acids Research 40: e21. 22127858 10.1093/nar/gkr908 PMC3273828

[12] Wang W, Bradley A, Huang Y (2009) A piggyBac transposon-based genome-wide library of insertionally mutated Blm-deficient murine ES cells. Genome research 19: 667–673. 19233961 10.1101/gr.085621.108 PMC2665785

[13] Diouf B, Cheng Q, Krynetskaia NF, Yang W, Cheok M, et al (2011) Somatic deletions of genes regulating MSH2 protein stability cause DNA mismatch repair deficiency and drug resistance in human leukemia cells. Nature Medicine 17: 1298–1303. 10.1038/nm.2430 PMC3192247 21946537

[14] Vora A, Mitchell CD, Lennard L, Eden TOB, Kinsey SE, et al (2006) Toxicity and efficacy of 6-thioguanine versus 6-mercaptopurine in childhood lymphoblastic leukaemia: a randomised trial. Lancet 368: 1339–1348. 17046466 10.1016/S0140-6736(06)69558-5

[15] Li MA, Pettitt SJ, Eckert S, Ning Z, Rice S, et al.. (2013) The piggybac transposon displays local and distant reintegration preferences and can cause mutations at non-canonical integration sites. Molecular and Cellular Biology. 10.1128/MCB.00670-12 PMC3624274 23358416

[16] Li MA, Turner DJ, Ning Z, Yusa K, Liang Q, et al (2011) Mobilization of giant piggyBac transposons in the mouse genome. Nucleic Acids Research 39: e148. 21948799 10.1093/nar/gkr764 PMC3239208

[17] Dantzer Fo, Amé J-C, Schreiber Vr, Nakamura J, Ménissier-de Murcia J, et al (2006) Poly(ADP-ribose) polymerase-1 activation during DNA damage and repair. Methods Enzymol 409: 493–510. 16793420 10.1016/S0076-6879(05)09029-4

[18] Murai J, Huang S-YN, Das BB, Renaud A, Zhang Y, et al (2012) Trapping of PARP1 and PARP2 by Clinical PARP Inhibitors. Cancer Research 72: 5588–5599. 23118055 10.1158/0008-5472.CAN-12-2753 PMC3528345

[19] Liu X, Han EK, Anderson M, Shi Y, Semizarov D, et al (2009) Acquired resistance to combination treatment with temozolomide and ABT-888 is mediated by both base excision repair and homologous recombination DNA repair pathways. Molecular cancer research 7: 1686–1692. 19825992 10.1158/1541-7786.MCR-09-0299

[20] Wang W, Bradley A, Huang Y (2009) A piggyBac transposon-based genome-wide library of insertionally mutated Blm-deficient murine ES cells. Genome Research 19: 667–673. 19233961 10.1101/gr.085621.108 PMC2665785

[21] Echeverri CJ, Beachy PA, Baum B, Boutros M, Buchholz F, et al (2006) Minimizing the risk of reporting false positives in large-scale RNAi screens. Nature Methods 3: 777–779. 16990807 10.1038/nmeth1006-777

[22] Liang Q, Conte N, Skarnes WC, Bradley A (2008) Extensive genomic copy number variation in embryonic stem cells. Proceedings of the National Academy of Sciences 105: 17453–17456. 10.1073/pnas.0805638105 PMC2582305 18988746

[23] Ramírez-Solis R, Davis AC, Bradley A (1993) Gene targeting in embryonic stem cells. Meth Enzymol 225: 855–878. 8231891 10.1016/0076-6879(93)25054-6

[24] Ying Q-L, Wray J, Nichols J, Batlle-Morera L, Doble B, et al (2008) The ground state of embryonic stem cell self-renewal. Nature 453: 519–523. 18497825 10.1038/nature06968 PMC5328678

[25] Yusa K, Zhou L, Li MA, Bradley A, Craig NL (2011) A hyperactive piggyBac transposase for mammalian applications. Proceedings of the National Academy of Sciences of the United States of America 108: 1531–1536. 21205896 10.1073/pnas.1008322108 PMC3029773

[26] Li MA, Pettitt SJ, Yusa K, Bradley A (2010) Genome-wide forward genetic screens in mouse ES cells. Methods in Enzymology 477: 217–242. 20699144 10.1016/S0076-6879(10)77012-9

[27] Kong J, Zhu F, Stalker J, Adams DJ (2008) iMapper: a web application for the automated analysis and mapping of insertional mutagenesis sequence data against Ensembl genomes. Bioinformatics 24: 2923–2925. 18974167 10.1093/bioinformatics/btn541 PMC2639305

[28] Li H, Durbin R (2009) Fast and accurate short read alignment with Burrows-Wheeler transform. Bioinformatics 25: 1754–1760. 19451168 10.1093/bioinformatics/btp324 PMC2705234

[29] Tutt A (2001) Mutation in Brca2 stimulates error-prone homology-directed repair of DNA double-strand breaks occurring between repeated sequences. The EMBO Journal 20: 4704–4716. 11532935 10.1093/emboj/20.17.4704 PMC125603

[30] Chen YT, Bradley A (2000) A new positive/negative selectable marker, puDeltatk, for use in embryonic stem cells. Genesis 28: 31–35. 11020714 10.1002/1526-968x(200009)28:1<31::aid-gene40>3.0.co;2-k

