# Supplementary material for: A Genetic Screen Using the PiggyBac Transposon in Haploid Cells Identifies Parp1 as a Mediator of Olaparib Toxicity
Source: PLoS One. 2013 Apr 25;8(4):e61520. doi: 10.1371/journal.pone.0061520 (PMC3636235; doi:10.1371/journal.pone.0061520)
Supplement: Protocol S1 — A detailed protocol for carrying out drug resistance screens. (DOCX) [file pone.0061520.s002.docx]

**Protocol S1: Drug resistance screens using haploid mouse embryonic stem cells**

**1. Mutagenesis**

1. Grow haploid ES cells in 2i+LIF medium on gelatin coated plates.
2. Harvest cells with trypsin or accutase and resuspend in medium at 10^6^ cells/ml with 20 µg/ml Hoechst 33342. Incubate at 37°C/5% CO_2_ for 20 minutes.
3. Sort haploid G1 cells using a FACS machine (e.g. BD FACSAria). Return cells to culture in 2i+LIF on an appropriately-sized culture plate.
4. Once sufficient haploid cells have been obtained, transfect 10^7^ cells per electroporation with 10 µg pCMV-hyPBase and 1 µg transposon donor plasmid. Electroporate at 270V, 500 µF in a 0.4 cm cuvette in a total volume of 800 µL PBS.
5. Each electroporation is plated immediately onto four wells of a six well plate, to make four libraries of independent insertion events. A separate plating at low density will allow estimation of the number of insertion events.
6. Begin selection in puromycin 24 hours post transfection.
7. Expand the libraries and freeze stocks and/or proceed to screens.

Growing cells in 2i medium is necessary to maintain the haploid population up to the point of mutagenesis. For screening, conventional serum containing ES cell medium could also be used; however cells have a tendency to become diploid in this medium.

**2. Determining drug concentration**

To isolate resistant mutants effectively, it is important to choose a drug concentration that kills all or nearly all wild type cells – this concentration may be quite high (µM range). This protocol is not suitable for drugs that only weakly kill ES cells. To determine the concentration to use, construct a clonogenic kill curve as follows:

1. Defrost a vial of HAP3 ES cells. Wash cells once in 5 mL medium to remove DMSO, and plate in 3 mL on one well of a six well plate.
2. Once confluent, passage to expand if necessary (calculate total number of cells required below). One confluent well should contain around 3 × 10^6^ cells.
3. Wash cells twice with PBS and add 500 µL of 0.25% trypsin-EDTA. Incubate at 37C for ten minutes or until cells have detached. Add 500 µL medium and pipette to a single cell suspension. Count cells, wash to remove trypsin and dilute to 5 × 10^5^ cells per mL in medium. Seed cells at 2.5 × 10^5^ cells per well on a 24-well gelatin coated plate by plating 500 µL of cell suspension per well.
4. Make dilutions of drug(s) to be tested in medium. For drugs on plate 10, starting concentrations can be obtained from these data. Otherwise, begin with 10-fold serial dilutions from 10 nM – 100 µM and use this as a starting point for a second experiment. For each concentration, 500 µL medium is required per day of drug treatment.
5. The day after plating cells, remove medium and replace with drug-containing medium. Growth media should be replaced daily. Treatment time depends on the drug, typically one to six days.
6. Continue growth until colonies are visible (8-10 days after plating). Wells treated with low concentrations will become very confluent and cells may begin to detach, but these are not of interest.
7. Rinse plates once with PBS and stain with methylene blue (1% in 70% ethanol) for 15 minutes, rinse with tap water and destain overnight.
8. Determine the lowest concentration that completely kills the cells. Ideally no surviving colonies, or only one or two (i.e SF < 10^-5^).

Example of stained plates:


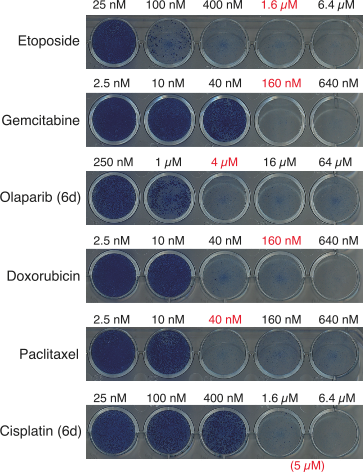


**3. Screening for resistant mutants**

Once a suitable concentration has been determined, this can be applied to the mutagenised cells in order to isolate resistant mutants. HAP3 wild type cells can be included as a control for cell killing.

1. Defrost library stock vials onto one well of a six well plate each as above.
2. Once confluent, split to six well plates for screening. One confluent well can be split 1:6 (i.e. to do six screens with about 5 × 10^5^ cells each), or passage further if more cells required (split 1:2 or 1:3 as needed).
3. Add drug medium the next day (2-3 mL medium required per day of treatment).
4. Change medium daily until colonies are visible.
5. Colonies will be ready to pick about 10 days after plating. Wash plates once with PBS, and add 3 mL PBS for picking. Add 50 µL trypsin solution to each well of a 96-well plate.
6. Pick colonies using the microscope and a 20 µL pipette set to 7-10 µL. Keep track of which library the colonies come from, and pick colonies with different morphologies (big/small/differentiated) so as not to miss anything.
7. Once the plate is full, incubate at 37°C for five minutes. Add 50 µL medium and pipette up and down to disrupt the colonies. Transfer to a 96-well flat bottomed plate,
8. Plates will be confluent after 3-5 days (allow enough time so that even the small colonies can expand). Change medium daily until most wells are turning yellow overnight.
9. Passage the cells to three replica plates. One plate can be re-treated with drug at this stage to provide a rough confirmation of the resistance phenotype.
10. Wash one of the replica plates twice with PBS and add 50 µL trypsin. Incubate at 37°C for ten minutes. Add 50 µL of serum-containing medium and pipette to break up clumps of cells. Add 100 µL of 2X freezing medium (20% DMSO, 20% FCS, 60% Knockout DMEM) and freeze overnight at -80°C in a polystyrene box. The final suspension can be divided into two plates to freeze in order to have a backup (or split 1:4 at step (i)). Matrix racks with 96 individual vials can also be used, so the whole plate doesn’t need to be defrosted when recovering mutants later (use a mineral oil overlay).
11. Wash the other plate with PBS and add 50 µL ES cell lysis buffer (100 mM NaCl, 50 mM Tris-HCl pH 7.5, 10 mM EDTA pH 8.0, 0.5% SDS. Add proteinase K fresh to 200 µg/ml). Place in a sandwich box with damp tissues and incubate overnight at 55°C for DNA preparation (the damp tissue prevents evaporation).

**4. Identifying insertion sites**

Insertion sites can be amplified and sequenced using the Splinkerette PCR method described in Li, M.A. et al. (2010) Genome-wide Forward Genetic Screens in Mouse ES Cells. *Methods in Enzymology* **477**:217-242 pp. 233-35
